# Meningococcal Septic Oligoarthritis: An Unusual Presentation Revealing Concurrent Multiple Myeloma

**DOI:** 10.7759/cureus.50555

**Published:** 2023-12-15

**Authors:** Luís A Rocha, Luciana Silva, João Miranda, Maria Inês Soares, Albina Moreira

**Affiliations:** 1 Internal Medicine, Centro Hospitalar Vila Nova de Gaia/Espinho, Vila Nova de Gaia, PRT

**Keywords:** invasive meningococcal disease, multiple myeloma, neisseria meningitidis, meningococcal arthritis, septic arthritis

## Abstract

Meningococcal invasive disease is rare in immunocompetent hosts but may occur in patients with risk factors. Septic arthritis is an uncommon form of presentation and is usually due to surgical colonization or hematogenous dissemination. We present a case of a 73-year-old woman, who recently underwent knee replacement surgery, presenting with right knee and left shoulder pain, swelling, and reduced range of motion. Antibiotic therapy was promptly initiated, and the identification of invasive meningococcal disease with septic arthritis was possible through blood cultures and synovial fluid analysis.

## Introduction

*Neisseria meningitidis* is a Gram-negative diplococcus that may be found as a benign commensal bacterium in the human nasopharynx in approximately 10% of healthy individuals [1-3]. In some cases, it can cause invasive diseases, such as bacteremia, meningitis, or septic arthritis. Mortality rates can be extremely high in untreated cases, reaching 80%, while treated cases range from 4 to 20% [1-3].

So far, twelve serogroups have been identified, six of which (A, B, C, W, X, and Y) are responsible for almost all cases of invasive meningococcal disease (IMD) [1-2]. A recent meta-analysis [2] quantified the significant risk of developing IMD in the presence of some factors, such as HIV infection, active or passive smoking, and crowded living environments. A Chinese meta-analysis recently estimated an annual incidence of 0.66-2.30 per 100,000 habitants [3].

To prevent the spread of resistant strains, it is of utmost importance to monitor antibiotic susceptibility regionally and globally [4].

Meningococcal septic arthritis (MSA) is an uncommon manifestation of IMD that occurs when *N. meningitidis* bacteria is responsible for the colonization of synovial joints. Colonization can also occur following surgery or penetrative trauma [5-11]. Primary MSA usually presents with mild symptoms such as localized pain, inflammation, and reduced range of movements of large joints. Several case reports [5-10] and a retrospective study [11] have been reporting several cases of monoarticular involvement. Despite being rarer, polyarticular presentations have also been reported [10-11]. Treatment of MSA typically involves arthroscopic washout or aspiration or surgical debridement and antimicrobial administration [5-11].

The occurrence of MSA is more frequent in immunocompromised patients. The association between IMD, particularly MSA, and the diagnosis of multiple myeloma is exceedingly rare, with only a few cases having been reported so far [12-14].

## Case presentation

We present the case of a 73-year-old woman who presented to the emergency department with right knee pain and swelling and reduced range of motion for the past four days. She had a medical history of hypertension and hypothyroidism. Additionally, she underwent knee replacement surgery three months prior on the right side.

On physical examination, she had a fever (body temperature of 38.7 degrees Celsius), blood pressure of 117/67mmHg, heart rate of 77 beats per minute, and oxygen saturation level of 98% at room air. The patient had an erythematous, warm, right knee with edema of the ipsilateral leg. Active and passive movements of the joint were greatly diminished because of intense pain. An erythematous maculopapular rash was noted on both legs and arms. The patient also had slight pain with active and passive movements of the left shoulder.

Initial blood tests revealed leucocytosis (16.970/uL) with neutrophilia (79%) and an elevated c-reactive protein level of 17.58 mg/dL and an erythrocyte sedimentation rate of 116 mm for the first hour. There was no elevated blood urea nitrogen (BUN), and liver function tests were unaffected (Table 1).

**Table 1:** Laboratory results

Lab result	Value	Reference range/unit
White blood cell count	16.97	3.6-11.0 (x 10E^3^/uL)
Neutrophil count	13.41	1.3-8.8 (x 10E^3^/uL)
C-reactive protein (CRP)	17.58	0-0.5 (mg/dl)
Erythrocyte sedimentation	116	0-20 (mm/Hr)
Urea	19	0.51-0.95 (mg/dL)
Serum immunoglobulins level		
Immunoglobulin G	6470.0	680-1450 (mg/dL)
Immunoglobulin A	<26.8	61-374 (mg/dL)
Immunoglobulin M	<18.2	40-248 (mg/dL)
Protein electrophoresis		
Albumina	3.1	4.2-5.1 g/dL
α1-Globulina	0.32	0.1-0.2 g/dL
α2-Globulina	0.81	0.5-0.8 g/dL
β-Globulina	0.66	0.5-0.9 g/dL
γ-Globulina	6.02	0.6-1.1 g/dL

**Figure 1:**
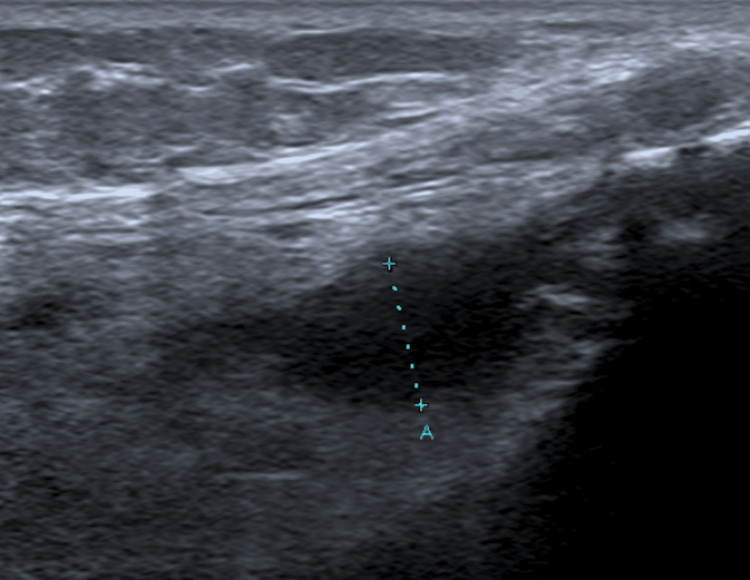
Right knee ultrasound Small-volume joint effusion measuring 5 mm in thickness is observed in the suprapatellar bursa, slightly more pronounced in the external recess of the joint cavity, where it measures 7 mm in thickness.

A right leg ultrasound showed articular effusion compatible with septic arthritis (Figure 1). The case was debated with the orthopedic team, and an articular ultrasound was performed to further characterize the effusion and guide drainage of articular fluid. However, the effusion was too small to perform a safe arthrocentesis. Blood cultures and serologic tests were collected, and empirical antibiotic therapy with ceftriaxone and vancomycin was started. The patient was admitted to the Internal Medicine Department.

During the initial days at the Internal Medicine department, the macular rash and fever resolved. Nevertheless, left shoulder pain got worse evolving with edema and motion limitation. Meanwhile, blood cultures allowed the detection of *N. meningitidis* bacteremia. The extensive serological panel was negative. The identification of IMD permitted antimicrobial treatment adjustment. To prevent the spread of meningococcal infection, droplet precautions and antimicrobial chemoprophylaxis in close contact were implemented.

**Figure 2:**
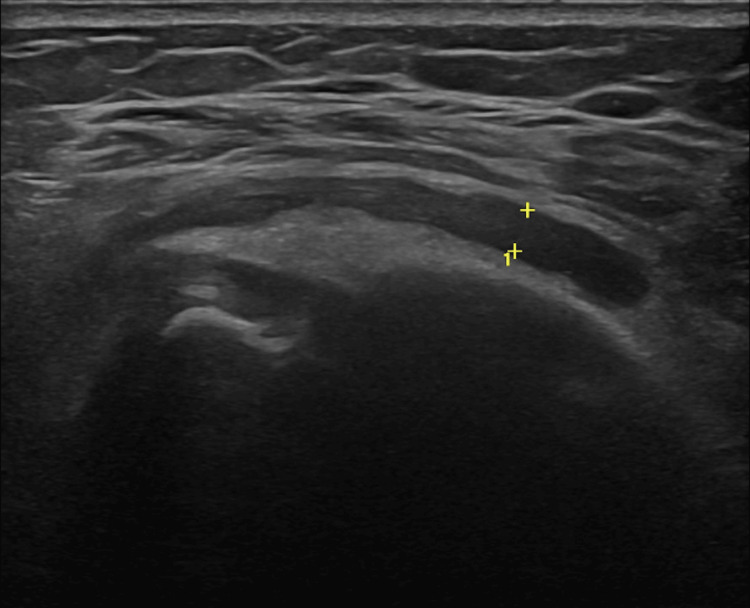
Left shoulder ultrasound Small joint effusion is observed with a maximum thickness of 4 mm, accompanied by some thin and sparse internal septa, suggesting inflammatory involvement.

Ultrasound-guided arthrocentesis of the left shoulder effusion (Figure 2) was performed, revealing a leucocyte count of 115,504/uL with 92.5% polymorphonuclear leucocytes. The culture of articular fluid, however, was negative.

Invasive meningococcal disease with polyarticular affection was assumed, and antimicrobial treatment was extended because of the presence of a knee prosthesis.

The presence of IMD in a previous immunocompetent host warranted further investigation of possible immunosuppressive disorders. Human immunodeficiency virus (HIV), Hepatitis B virus (HBV), and Hepatitis C virus (HCV) serologies were negative. However, her serum immunoglobulin G level was high at 6,470.0 (mg/dL), and protein electrophoresis revealed a monoclonal G peak (6.02 g/dL). Urine immunofixation confirmed the detection of a monoclonal immunoglobulin G/lambda, with a value of 6.61 mg/dL, indicating a significant presence of M protein.

The patient evolved favorably with a resolution of fever, reduction of inflammatory markers, and joint mobility recovery. Six weeks later, the patient underwent a surgical intervention to explant right knee prosthesis and debridement. A bone biopsy performed during surgery confirmed multiple myeloma.

The patient underwent four weeks of antibiotic therapy in preparation for a second surgical intervention, where a new knee prosthesis was implemented. 

## Discussion

*N. meningitidis *infection occurs rarely in immunocompetent hosts, but, in some cases, it can cause invasive diseases such as bacteremia or septic arthritis. The risk of developing invasive meningococcal disease is greater in HIV-infected patients, active or passive smokers, and people living in crowded areas [1-4].

The initial clinical picture raised suspicion of an infection of the right knee prosthesis. Unfortunately, the risk of draining knee synovial fluid and obtaining early microbiological cultures delayed the identification of the bacterial agent. Meningococcal septic arthritis can occur either by direct colonization during surgery or trauma or by secondary colonization in patients with bacteremia [5-11]. Considering the recent knee surgery, the most probable scenario is that the source of this infection was knee prosthesis colonization, with later bacteremia and ultimately hematogenous dissemination to the left shoulder. Microbiological cultures performed after left shoulder fluid drainage were negative most likely because of antimicrobial therapy already in progress for several days.

During the etiological investigation of possible immunosuppressive disorders, we identified a monoclonal immunoglobulin G/lambda peak. Later, the bone biopsy confirmed the diagnosis of multiple myeloma. This finding may help explain why this patient with no known risk factors developed invasive meningococcal disease.

## Conclusions

Microbiological cultures performed after draining fluid from the left shoulder were negative, most likely due to the ongoing antimicrobial therapy for several days. The identification of specific bacteria, such as *N. meningitidis*, warrants further investigation that can lead to an early diagnosis of hidden conditions with a significant prognostic impact.
